# Supplementary material for: Structural basis for assembly of vertical single β-barrel viruses
Source: Nat Commun. 2019 Mar 12;10:1184. doi: 10.1038/s41467-019-08927-2 (PMC6414509; doi:10.1038/s41467-019-08927-2)
Supplement: Supplementary file 1 — Supplementary Information [file 41467_2019_8927_MOESM1_ESM.pdf]

SUPPLEMENTARY INFORMATION FOR

Structural basis for assembly of vertical single β-barrel viruses

Isaac Santos-Pérez[1,5], Diego Charro[1,5], David Gil-Carton[1], Mikel Azkargorta[2], Felix Elortza[2], Dennis H Bamford[3], Hanna M Oksanen[3], Nicola G A Abrescia[1,4,*]

[1]Molecular Recognition and Host-pathogen Interactions Programme, CIC bioGUNE, CIBERehd, Bizkaia Technology Park, 48160 Derio, Spain. [2] Proteomics Platform, CIC bioGUNE, CIBERehd, ProteoRed-ISCIII. [3]Molecular and Integrative Biosciences Research Programme, Faculty of Biological and Environmental Sciences, Viikki Biocenter, University of Helsinki, P.O. Box 56, Viikinkaari 9, 00014 Helsinki, Finland. [4]IKERBASQUE, Basque Foundation for Science, 48013 Bilbao, Spain.

[5]These authors contributed equally to this work

[*]Correspondence: nabrescia@cicbiogune.es

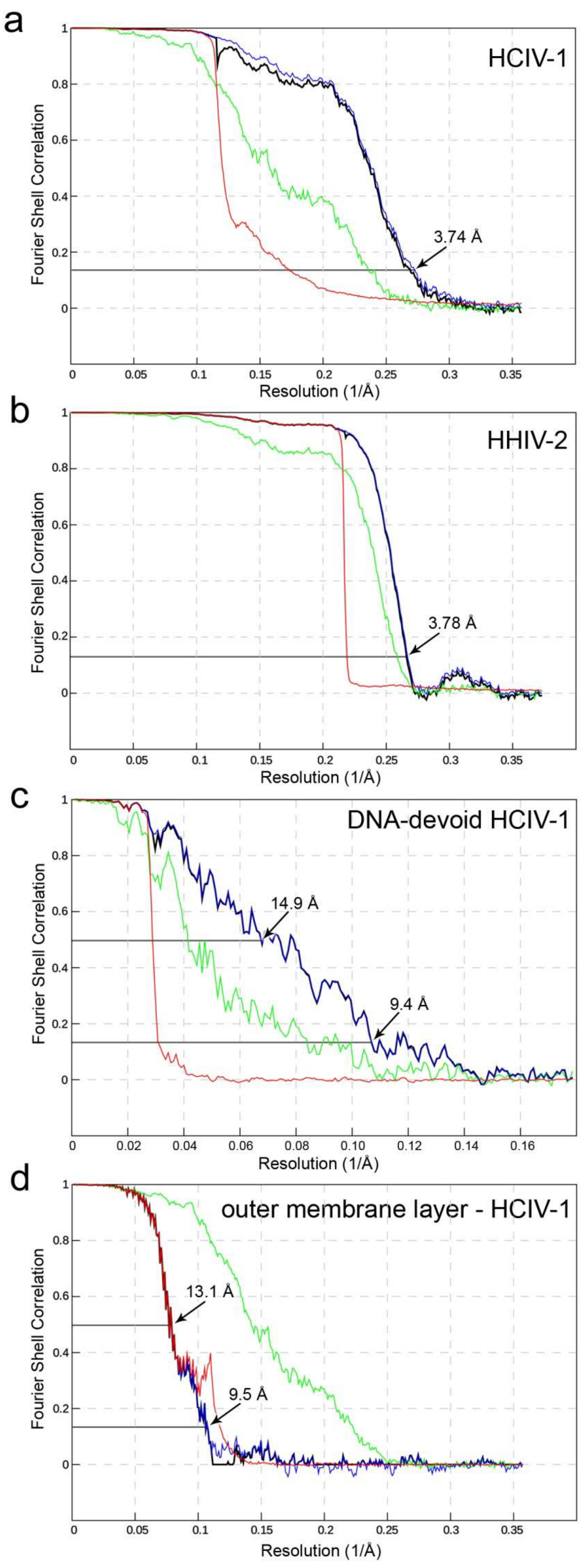
a
HCIV-1
3.74 Å
Fourier Shell Correlation
Resolution (1/Å)
b
HHIV-2
3.78 Å
Fourier Shell Correlation
Resolution (1/Å)
c
DNA-devoid HCIV-1
14.9 Å
9.4 Å
Fourier Shell Correlation
Resolution (1/Å)
d
outer membrane layer - HCIV-1
13.1 Å
9.5 Å
Fourier Shell Correlation
Resolution (1/Å)

Supplementary Figure 1. **Fourier-Shell-Correlation of the reconstructed particles** **a**, FSC curve of HCIV-1 with reported resolution of 3.74 Å at the 0.143 criterion (black line: corrected map). Other colored curves represent FSC for: blue line, masked maps; red line, phase randomized masked maps; green line, unmasked maps; (same colour scheme holds for the remaining panels). **b**, FSC curve of HHIV-2 with reported resolution of 3.78 Å at the 0.143 criterion. **c**, FSC of DNA-devoid HCIV-1 particles with reported resolution of ~15 Å at the more conservative 0.5 criterion as few number particles contributes to the binned 3D reconstruction. **d**, FSC curves (black, blue and red lines) correspond to the outer leaflet of the membrane vesicle of HCIV-1 with reported resolution of ~13 Å at the 0.5 criterion; green line FSC corresponds to the unmasked maps and therefore the whole virion.

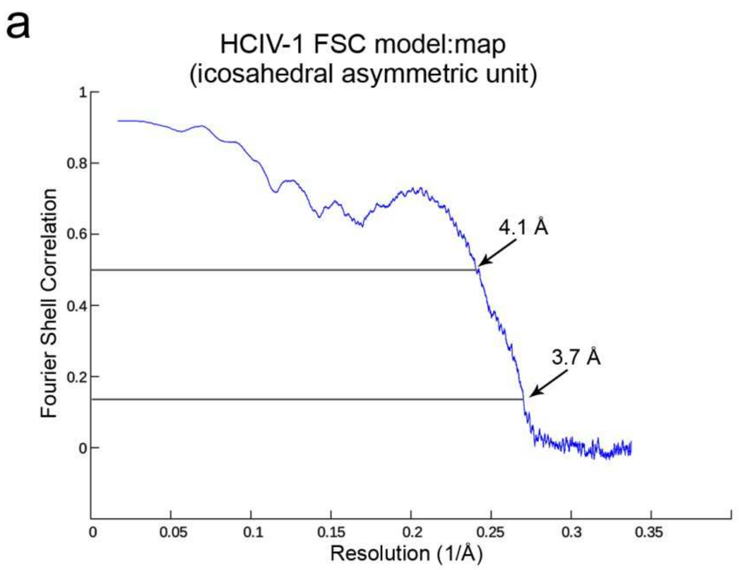

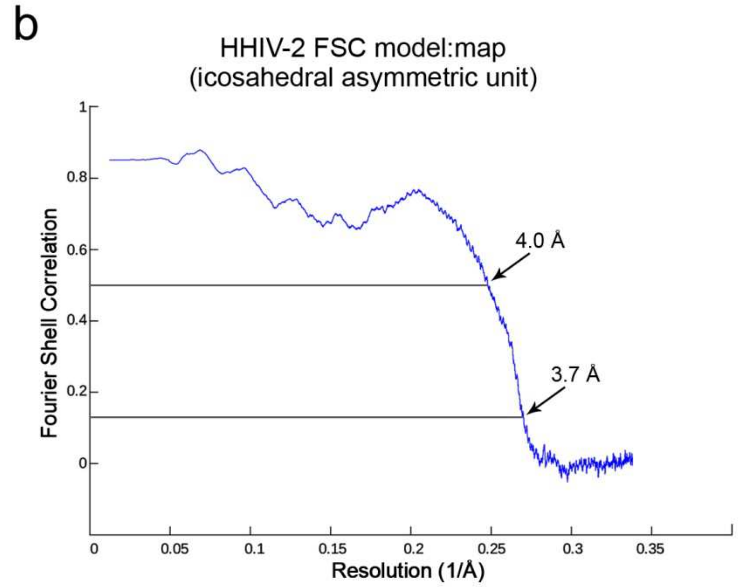

Supplementary Figure 2. Fourier-Shell-Correlation model:map for IAU models
a, Masked FSC calculated between the atomic model of the Icosahedral Asymmetric Unit (IAU) of HCIV-1 (composed of twelve copies of VP4, fifteen copies of VP7, one copy of VP9, one and half copies of GPS-II, one copy of GPS-III and one copy of an unknown protein fragment; the latter three proteins as ALA-models) and the corresponding boxed map using phenix.mtriage[1]. **b**, As top but for the IAU of HHIV-2 (composed of twelve copies of VP4, fifteen copies of VP7, one copy of VP9, two copies of GPS-III, one copy of VP16 and one copy of an unknown polypeptide chain; the latter three proteins as ALA-models). In both HCIV-1 and HHIV-2 viruses 3.7 Å is the resolution cutoff up at 0.143 to which the model and map Fourier coefficients are similar; the FSC at 0.5 is also reported.

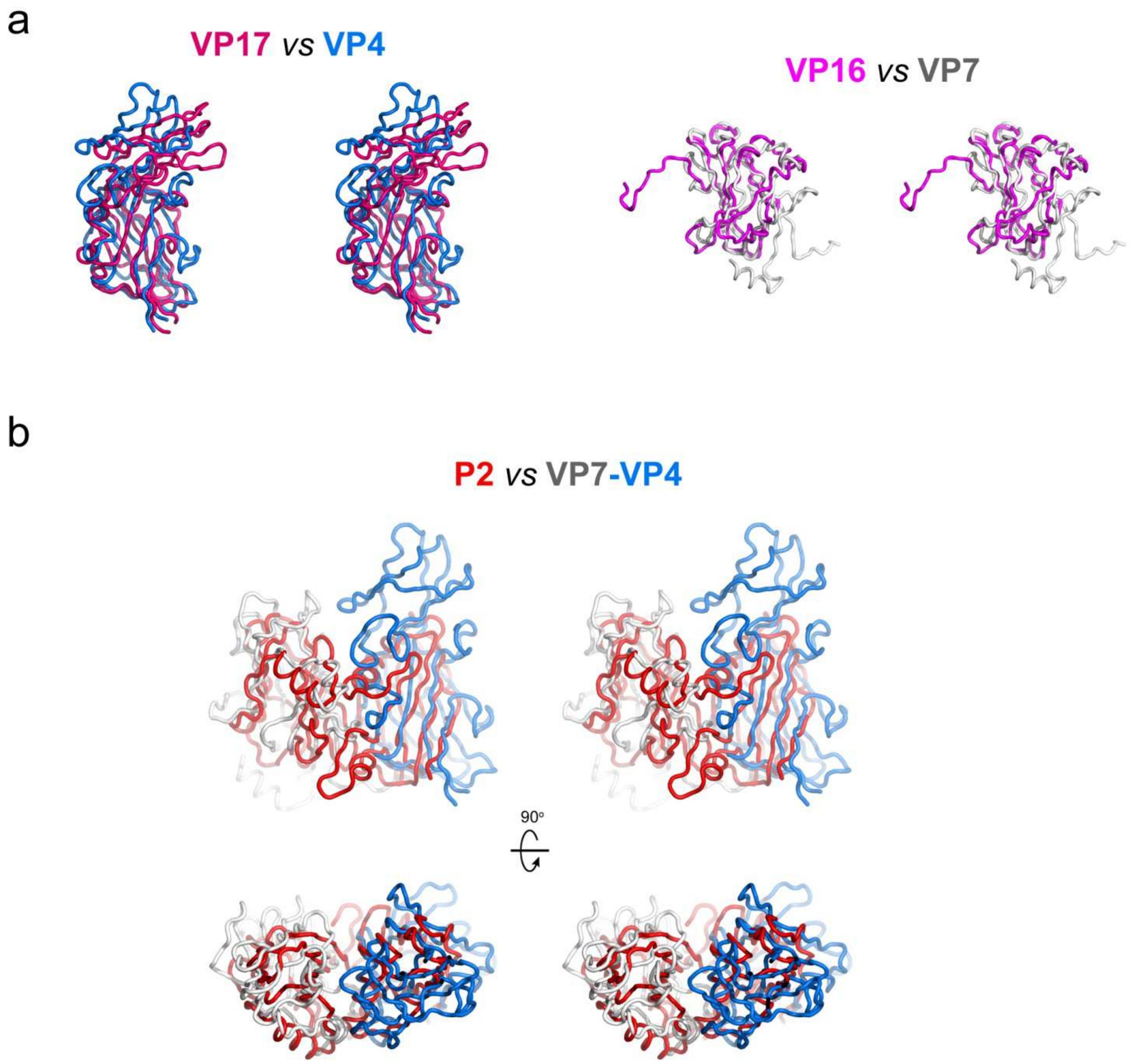

Supplementary Figure 3. Superimposition of the MCPs across the single vertical β-barrel and double vertical β-barrel proteins

a, Left, stereoview of *Thermus* phage MCP VP17 (dark-magenta; 232 residues out of 291 modeled) (PDB ID 3ZN6; https://www.rcsb.org/structure/3ZN6) superimposed onto HCIV-1 MCP VP4 (marine-blue; subunit A in Fig.1 centre, 229 residues out of 232 modeled), 3.2 Å rmsd, 194 Cα equivalences; right as left but with *Thermus* phage MCP VP16 (magenta; 150 residues out of 173 modeled) (PDB ID 3ZN6) onto HCIV-1 MCP VP7 (light-grey; subunit D in Fig. 1 centre, 174 residues out of 184 modeled), 2.1 Å rmsd, 115 Cα equivalences. **b,** Stereoviews of HCIV-1 MCP VP7-VP4 heterodimer (403 residues) onto the double β-barrel MCP P2 of bacteriophage PM2 (red; 269 residues, PDB ID 2VVF; https://www.rcsb.org/structure/2VVF), 4.2 Å rmsd with 189 Cα equivalences; the relative angular orientation between the individual HCIV-1 VP7-VP4 β-barrels practically replicates that of double β-barrel MCP.

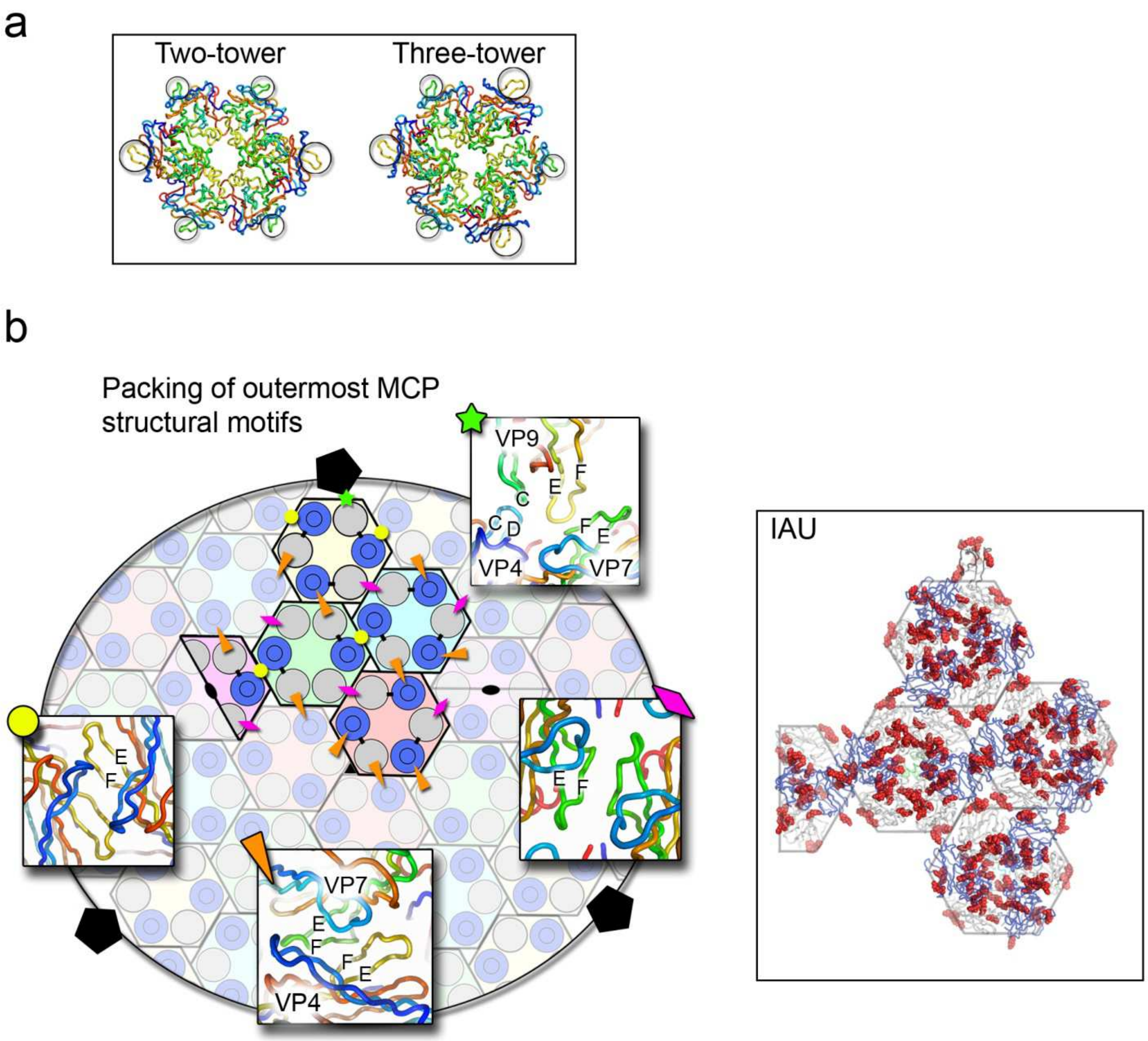

Supplementary Figure 4. Packing of MCPs VP4 and VP7 in HCIV-1
a, Two-tower and three-tower capsomers viewed along the pseudo-two and three-fold axes and represented as cartoon tube colored in rainbow from blue (N-terminus) to red (C-terminus); black-circles mark the outermost regions and connecting loops involved in MCPs packing within and across the IAU (GPS-II and III proteins omitted for clarity). **b**, Left, schematic representation of the IAU in the context of the neighboring capsomers with packing motifs depicted by a yellow circle: pseudo two-fold related VP4 residues 28-34 (leading to strand C) and 148-166 (in strand F); a magenta rhomboid: pseudo two-fold related VP7 residues 81-87 (connecting strands E-F); an orange triangle: VP7 residues 31-34 with VP4 residues 33-36 and VP7 residues 81-89 with VP4 residues 156-161 (leading to corresponding strands F); the green star marks the location of interaction between protein VP9 and adjacent peripentonal VP7-VP4 heterodimers. Insets depict, viewed from the top, the spatial organization of these motifs. Right, location of ARG residues as red-spheres within the IAU, some of them at the periphery of the capsomer are conceivably involved in H-bonds and salt-bridges across subunits, *eg.* VP4 ARG33 with VP7 ASP31 (orange triangle); MCPs VP4 and VP7 displayed as in Fig. 1c, semi-transparent, and capsomers outlined by grey hexagons and half-hexagon.

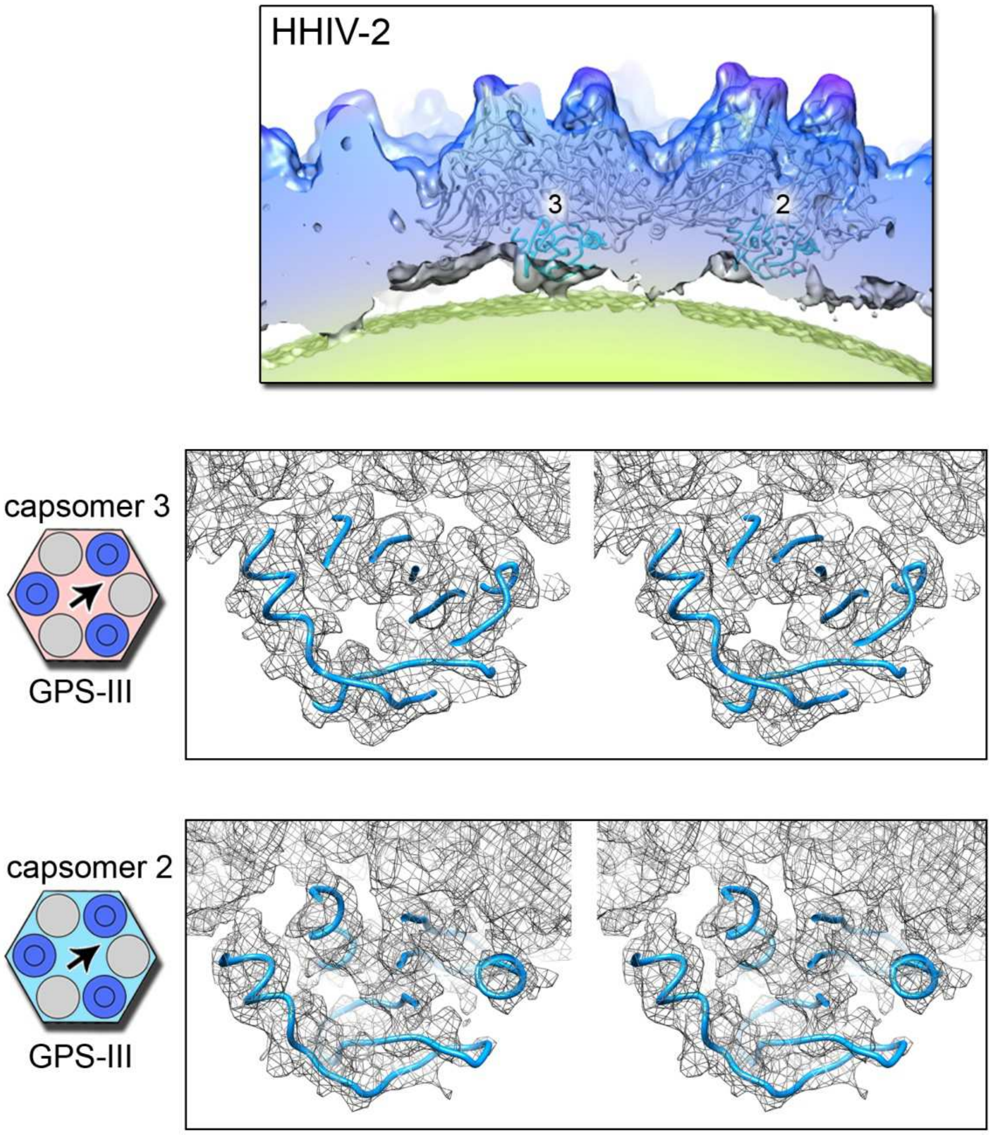

Supplementary Figure 5. HHIV-2 GPS-III proteins
Top, cut-through of the filtered cryo-EM map of HHIV-2 color-coded as Fig. 1a right with cartoon tube of the IAU model with numbers marking capsomers Nos. 2 and 3 as Fig. 1a centre showing their proximity to the membrane outer leaflet (OL). Centre and bottom, wall-eyed stereoview of HCIV-1 GPS-III poly-ALA (cyan) model fitted into the HHIV-2 density (mesh grey) underneath capsomers Nos. 3 and 2 respectively, differently oriented (the density underneath capsomer 2 is weaker than that underneath capsomer 3). The density protruding from the helix in the center panel identifies the presence of large side-chains, also clearly visible in the corresponding HCIV-1 density region.

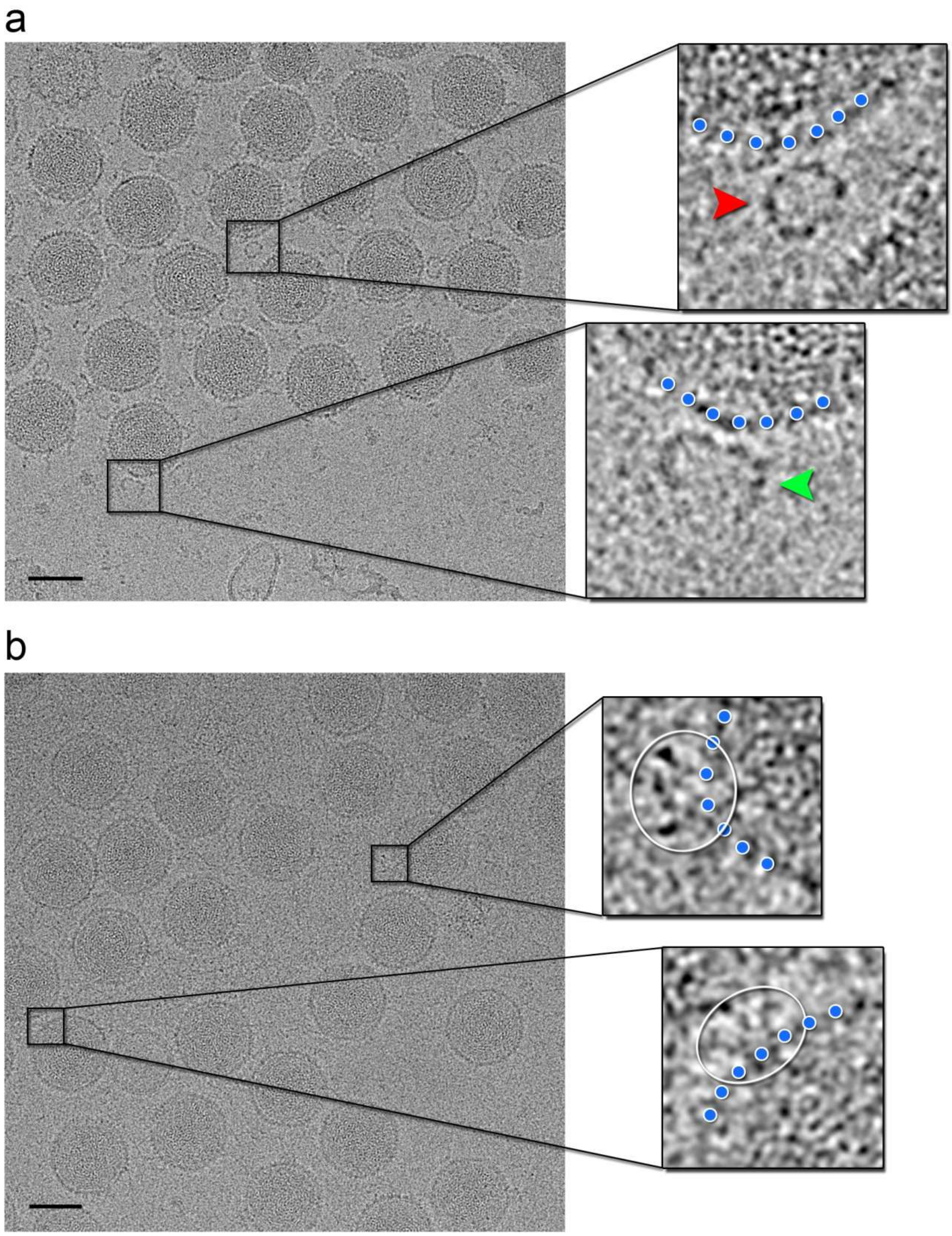

Supplementary Figure 6. Raw images of vertex complexes

a, Overall view of a 2D cryo-image of HCIV-1 sample (estimated defocus: -2.5 μm) with black square marking some of the vertex complexes. Insets, enlarged views of the dimeric vertex complex in close- (red arrow) and open-conformation (green arrow) respectively, blue dots schematically mark the capsid profile; insets have been gaussian blurred to facilitate their recognition. See also Supplementary Figure 7 and Movie 1. **b**, As (**a**) but for HHIV-2 (estimated defocus: -1.5 μm) in this case the vertex complex is pentameric, see Supplementary Figure 8. Scale bar, 50 nm.

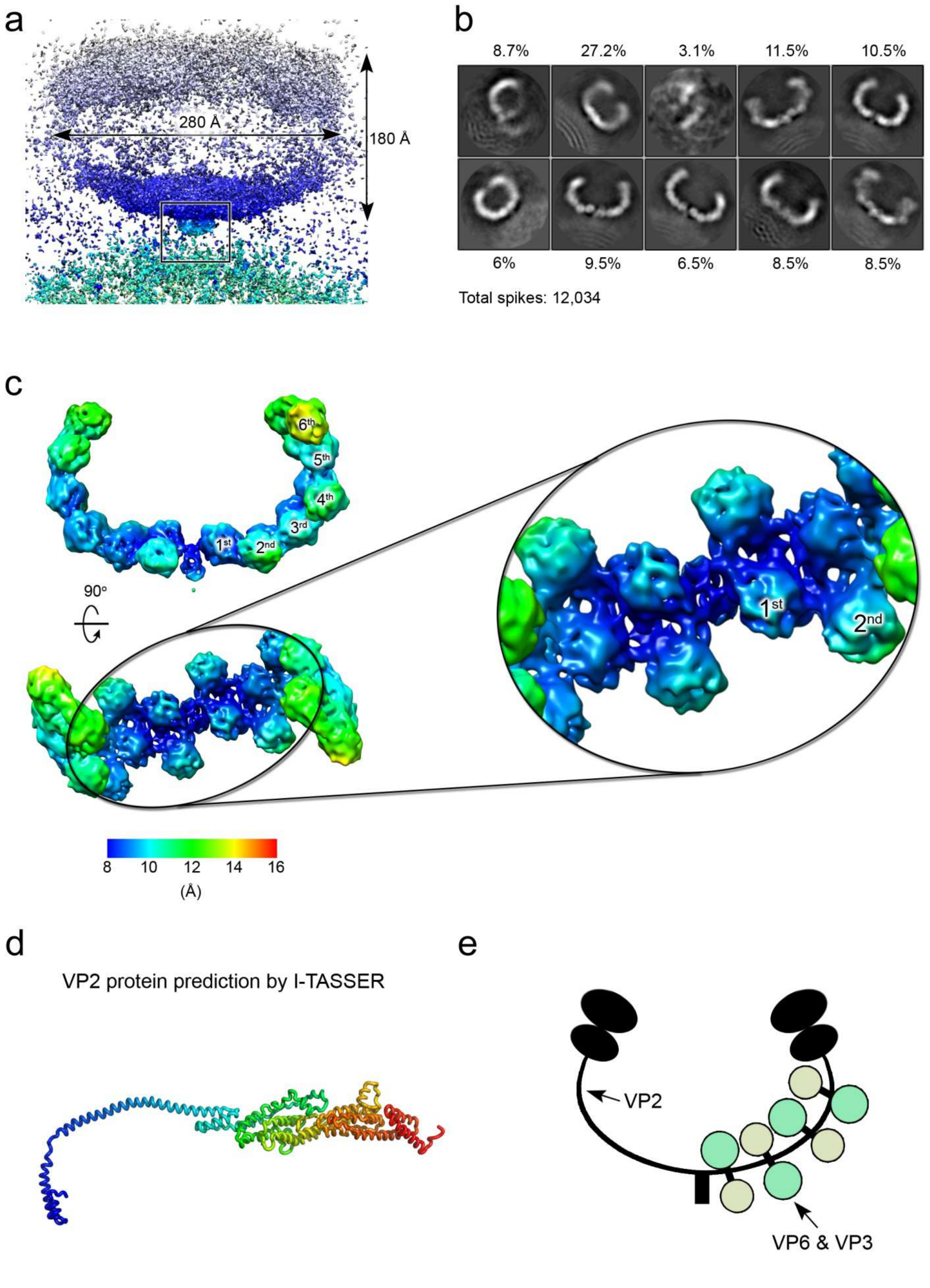
a
280 Å
180 Å
b
8.7%
27.2%
3.1%
11.5%
10.5%
6%
9.5%
6.5%
8.5%
8.5%
Total spikes: 12,034
c
6th
5th
4th
3rd
2nd
1st
90°
8
10
12
14
16
(Å)
d
VP2 protein prediction by I-TASSER
e
VP2
VP6 & VP3

Supplementary Figure 7. HCIV-1 vertex complex

a, Overall view of the HCIV-1 spike before localized reconstruction; black rectangle marks the density corresponding to the region stemming from the penton to the 'horn' – site of the symmetry mismatch (color-coded as in Fig. 1a left). **b**, Ten distinct classes of the dimeric horn-like spikes attached to the five-fold vertices showing their structural flexibility; with percentages indicating the class abundance over the total number of spikes analysed. **c**, Top, view of the 3D reconstructed spike by localized methods[2] orthogonal to the two-fold axis showing its architecture as a pearl collar necklace composed by six layers (labeled from 1st to 6th); bottom, view of the dimeric spike along the two-fold axis displaying the arrangement of globular protein domains as shown in the oval inset (right); the density has been colored accordingly to the local-resolution estimation (see the key below). **d**, Possible model from I-TASSER server for the spike protein VP2 predicting an elongated structure (estimate of the confidence of structure prediction C-score = -1.15; typically from -5 to 2, higher the better[3]] with the template structure being PDB ID 5YFP (https://www.rcsb.org/structure/5yfp; chain F) and with whom VP2 shares 6.4% sequence identity in the structurally aligned region. **e**, Proposed schematic architecture of the HCIV-1 spike complex with VP2 forming the dimeric scaffolding horns with globular VP3 and VP6 (corresponding models were predicted by I-TASSER but with very low confidence) arranging along its length; this protein arrangement could also extend to the SH1 spike complex[4].

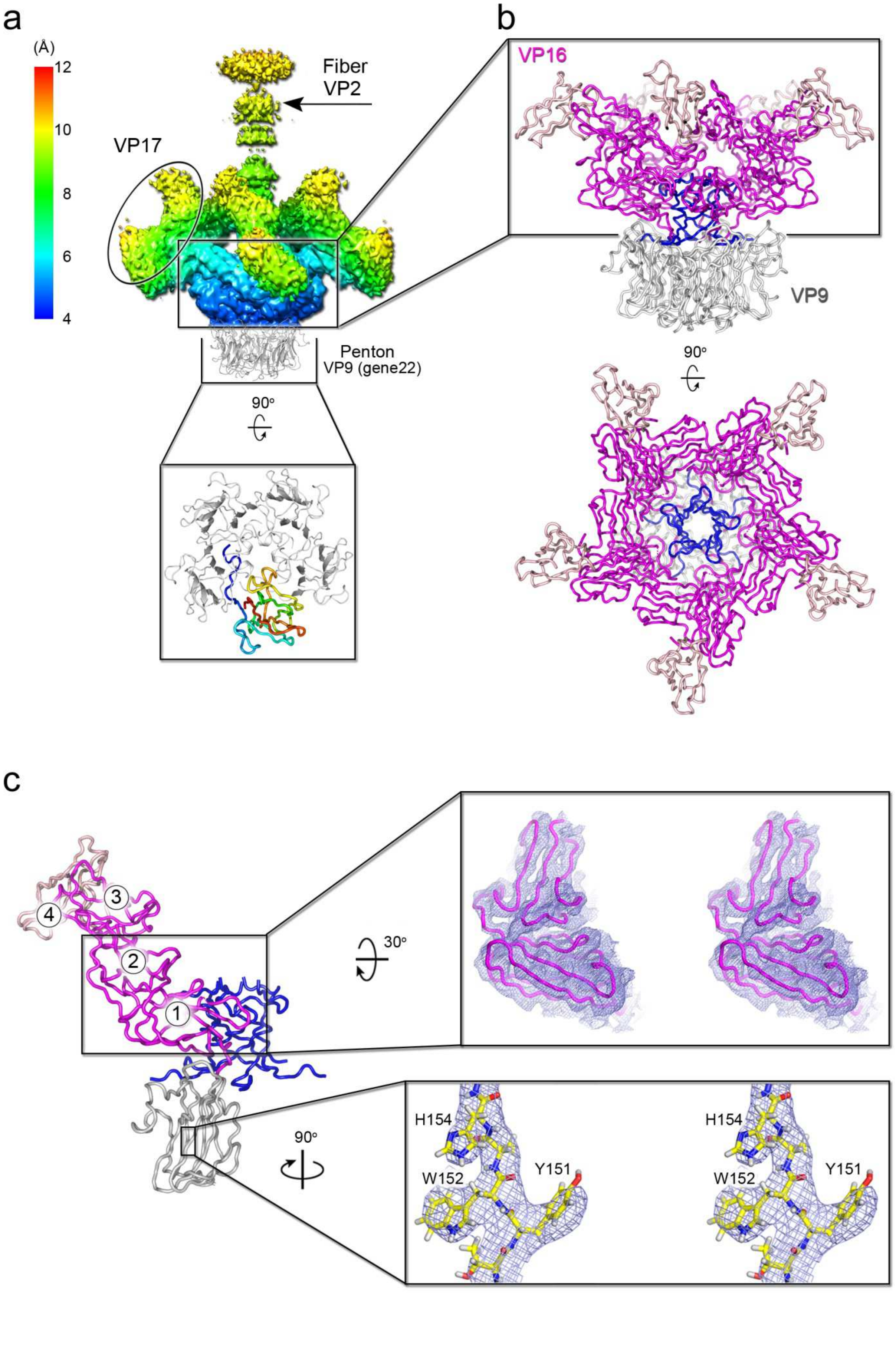
a
(Å)
12
10
8
6
4
Fiber
VP2
VP17
Penton
VP9 (gene22)
90°
b
VP16
VP9
90°
c
1
2
3
4
30°
90°
H154
W152
Y151
H154
W152
Y151

Supplementary Figure 8. HHIV-2 vertex complex.

a, Local resolution estimation[5] of the HHIV-2 spike complex density (resolution key on the left) with the penton represented in whitesmoke cartoon viewed perpendicularly to the icosahedral five-fold axis; regions corresponding to the putatively assigned fiber VP2 and VP17 are marked by a black arrow and oval respectively while the region assigned to the five copies of VP16 is defined by the black rectangle. The below inset shows a magnified view of the penton along the five-fold axis with one VP9 subunit colored from N-terminal (blue) to C-terminal (red) – the first visible N-terminal residues, about ten, clamp the base of the adjacent jelly-roll penton subunit. **b**, Top, poly-ALA model manually built and assigned to VP16 (about 217 residues modeled out of 253; see Methods) represented as magenta cartoon tube while in pink a further possible jelly-roll fragment putatively belonging to VP17; in the center in blue cartoon tube five copies of a poly-ALA stretch that coaxially cements VP16, VP9 (grey cartoon) and the central fiber (no models for VP2 and VP17 could be reliably traced); bottom, as above but viewed from the top to highlight the propeller-like appearance reminiscent of STIV and PM2 spike complexes[6,7]. **c**, As (**b**) top, multidomain composition of VP16 (tube magenta; numbers 1-3 mark the jelly-roll domains) and its arrangement relative to a possible VP17 domain (tube pink and labeled as 4), to the penton subunit VP9 (light-grey) and to the five copies of the polypeptide stretch (blue). The insets on the right show in wall-eye stereoview the densities (light-blue mesh) for the VP16 domains 1 and 2 closer to the vertex centre (top) and for the VP9 single β-barrel that has permitted the registering of the sequence (bottom).

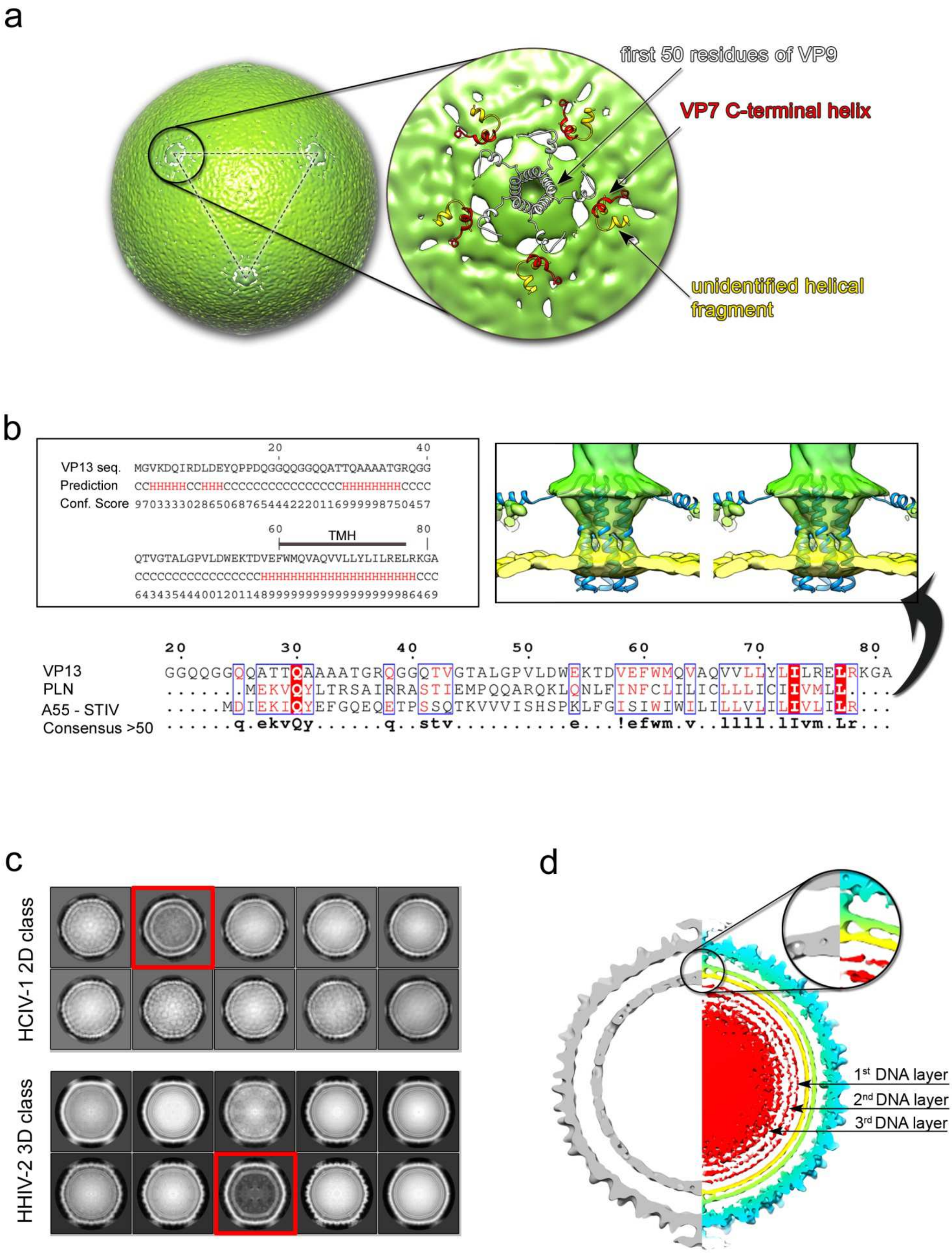
a
first 50 residues of VP9
VP7 C-terminal helix
unidentified helical fragment
b
VP13 seq. MGVKDQIRDLDEYQPPDQGGQQGGGQQATTQAAAATGRQGG
Prediction CCHHHHHCCHHHCCCCCCCCCCCCCCCCHHHHHHHHCCCC
Conf. Score 9703333028650687654442220116999998750457
TMH
QTVGTALGPVLDWEKTDVEFWMQVAQVVLLYLILRELRKGA
CCCCCCCCCCCCCCCCCHHHHHHHHHHHHHHHHHHHHHHCCC
64343544400120114899999999999999999986469
VP13 GGQQGGQQATTQAAAATGRQGGQTVGTALGPVLDWEKTDVEFWMQVAQVVLLYLILRELRKGA
PLN .......MEKVQYLTRSAIRRASTIEMPQQARQKLQNLFINFCLILICLLLICIIVMLL....
A55 - STIV .....MDIEKIQYEFGQEQETPSSQTKVVVISHSPKLFGISIWIWILILLVLILIVLILR...
Consensus >50 ......q.ekvQy......q..stv..........e...!efwm.v..llll.lIvm.Lr...
c
HCIV-1 2D class
HHIV-2 3D class
d
1st DNA layer
2nd DNA layer
3rd DNA layer

Supplementary Figure 9. Membrane vesicle and DNA-devoid procapsid

a, Surface rendering (light-green) of the outer leaflet (OL) of the membrane vesicle of HCIV-1 (cryo-EM map filtered to 9.5 Å as from Supplementary Figure 1d) displayed in Chimera. Inset, enlarged area of the density of the OL beneath the penton viewed along the five-fold axis (inner leaflet and genome removed for clarity) with some ordered structural elements populating the region around the five-fold and in between the capsid shell and the OL. **b**, Top left, secondary structure prediction of VP13 using I-TASSER with marked the transmembrane helix segment (TMH) as from phobious webserver (http://phobius.sbc.su.se/); coil as C, helix as H, transmembrane helix as TMH, and the confidence score from 1 to 9, with 9 being the highest. Bottom, multiple sequence alignment between VP13, human phospholamban (PLN) and STIV A55 with red box, white characters as strict identities and with white box, red characters as similarities and with the consensus sequence > 50% marked (Espript software[8]). Top right, stereoview of cryo-EM density (gaussian filtered with a 2.1 Å width in Chimera) corresponding to the membrane complex below the VP9 penton with fitted in as rigid-body with the pentameric structure of PLN (PDB ID 2M3B; https://www.rcsb.org/structure/2M3B) showed as cartoon. **c**, 2D and 3D classification for HCIV-1 (top) and HHIV-2 (bottom) particles respectively, and identification of DNA-devoid particles with the corresponding class marked by a red square. **d**, Side-by-side comparison of central sections (14 Å thick) of DNA devoid HCIV-1 particles (left, grey density) and DNA packaged particles (right, cryo-EM map filtered at 9.5 Å and color-coded as Fig. 1a) with black-arrows indicating the 1st, 2nd and 3rd layer of the DNA with the inset showing the clear expansion of the membrane when the DNA is packaged.

Supplementary Table 1. Cryo-EM data collection, refinement and validation statistics for HCIV-1

**Data collection**				
Nominal magnification	59,000			
Voltage (kV)	300			
Electron exposure ($e^-/Å^2$)	36			
Defocus range (µm)	0.6-3.9			
Sampling interval (Å/pixel)	1.4			
Frames	27			
N° micrographs	3,218			
**Cryo-EM processing**				
	Full virus	*DNA-devoid virus*	*Horn-like spike-1*	*Horn-like spike-2*
Contributing particles	3,414	84	11,607	9,720
Box Size (pixel)	768	384	300	300
Pixel size (Å)	1.4	2.8	1.4	1.4
Symmetry	I1	I1	C2	C2
Map resolution (Å)<br>FSC threshold	3.74<br>0.143	14.9<br>0.5	8.9<br>0.143	18.3<br>0.143
Map sharpening *B* factor ($Å^2$)	-40	0	-640	-3213
EMDB code	0174	0050	0073	0072
**Structure refinement and model validation**				
	Full virus model†			
PDB ID	6H9C			
CC, volume	0.761			
CC, around masked atoms	0.763			
Model resolution (Å)	3.74			
Model composition<br>Non-hydrogen atoms<br>Protein residues	 2,621,580<br>343,679			
B factors ($Å^2$)<br>Protein	 105.9			
R.m.s. deviations<br>Bond lengths (Å)<br>Bond angles (°)	 0.01<br>1.26			
Validation<br>MolProbity score<br>All-Atoms Clashscore<br>Poor rotamers (%)	 1.95<br>5.68<br>0.00			
Ramachandran plot<br>Favored (%)<br>Allowed (%)<br>Disallowed (%)	 85.80<br>14.20<br>0.00			
EMRinger score	1.84			
FSC (map, model map) threshold=0.143	3.70*			
Map resolution estimate d99 metric (Å)	3.94*			

†*The icosahedral asymmetric unit (IAU) of the full virus model is composed of twelve copies of VP4, fifteen copies of VP7, one copy of VP9, one and half copies of GPS-II, one copy of GPS-III and one copy of an unknown protein fragment (the latter three proteins as ALA-models).*

**These values have been calculated using phenix.mtriage*[1] *on the IAU and corresponding boxed map.*

Supplementary Table 2. Cryo-EM data collection, refinement and validation statistics for HHIV-2

**Data collection**		
Nominal magnification	60,000	
Voltage (kV)	300	
Electron exposure ($e^{-}/Å^2$)	35	
Defocus range (μm)	0.7-3.0	
Sampling interval (Å/pixel)	1.34	
Frames	7	
N° micrographs	2,786	
**Cryo-EM processing**		
	Full virus	*Vertex complex*
Contributing particles	11,446	58,176
Box Size (pixel)	760	300
Pixel size (Å)	1.34	1.34
Symmetry	I1	C5
Map resolution (Å)<br>FSC threshold	3.78<br>0.143	7.7<br>0.143
Map sharpening *B* factor ($Å^2$)	-40	-322
EMDB code	0172	0131
**Structure refinement and model validation**		
	Full virus model††	
PDB ID	6H82	
CC, volume	0.758	
CC, around masked atoms	0.758	
Model resolution (Å)	3.8	
Model composition<br>Non-hydrogen atoms<br>Protein residues	 2,658,480<br>354,360	
B factors ($Å^2$)<br>Protein	 97.9	
R.m.s. deviations<br>Bond lengths (Å)<br>Bond angles (°)	 0.009<br>1.26	
Validation<br>MolProbity score<br>All-Atoms Clashscore<br>Poor rotamers (%)	 1.74<br>4.51<br>0	
Ramachandran plot<br>Favored (%)<br>Allowed (%)<br>Disallowed (%)	 89.36<br>10.62<br>0.02	
EMRinger score	1.55	
FSC (map, model map) threshold=0.143 (Å)	3.71*	
Map resolution estimate d99 metric (Å)	4.16*	

††*The IAU of the full virus model is composed of twelve copies of VP4, fifteen copies of VP7, one copy of VP9, two copies of GPS-III, one copy of VP16 (three out of four domains) and one copy of an unknown polypeptide chain (the latter three proteins as ALA-models).*

**These values have been calculated using phenix.mtriage[1] on the IAU and corresponding boxed map.*

SUPPLEMENTARY REFERENCES

1 Afonine, P. V., Headd, J. J., Terwilliger, T. C. & Adams, P. D. New tool: phenix.real_space_refine. *Comput. Crystallogr. Newsl.* **4**, 43-44 (2013).

2 Ilca, S. L. *et al.* Localized reconstruction of subunits from electron cryomicroscopy images of macromolecular complexes. *Nat. Commun.* **6**, 8843, (2015).

3 Yang, J. *et al.* The I-TASSER Suite: protein structure and function prediction. *Nat. Methods* **12**, 7-8, (2015).

4 Jaalinoja, H. T. *et al.* Structure and host-cell interaction of SH1, a membrane-containing, halophilic euryarchaeal virus. *Proc. Natl. Acad. Sci. U S A* **105**, 8008-8013, (2008).

5 Scheres, S. H. RELION: implementation of a Bayesian approach to cryo-EM structure determination. *J. Struct. Biol.* **180**, 519-530, (2012).

6 Veesler, D. *et al.* Atomic structure of the 75 MDa extremophile Sulfolobus turreted icosahedral virus determined by CryoEM and X-ray crystallography. *Proc. Natl. Acad. Sci. U S A* **110**, 5504-5509, (2013).

7 Abrescia, N. G. *et al.* Insights into virus evolution and membrane biogenesis from the structure of the marine lipid-containing bacteriophage PM2. *Mol. Cell* **31**, 749-761, (2008).

8 Robert, X. & Gouet, P. Deciphering key features in protein structures with the new ENDscript server. *Nucleic Acids Res.* **42**, W320-324, doi:10.1093/nar/gku316 (2014).
