# Supplementary material for: Structural basis for assembly of vertical single β-barrel viruses
Source: Nat Commun. 2019 Mar 12;10:1184. doi: 10.1038/s41467-019-08927-2 (PMC6414509; doi:10.1038/s41467-019-08927-2)
Supplement: Supplementary file 3 — Description of Additional Supplementary Files [file 41467_2019_8927_MOESM3_ESM.pdf]

Description of Additional Supplementary Files

File Name: Supplementary Movie 1
Description: Flexibility of the HCIV-1 vertex complex. Morphing between the two 3D reconstructed conformations of the HCIV-1 vertex complex (green, open conformation; red, close conformation).
